# Predatory publishers and fraudulent conferences: Perspectives and implications for novice researchers

**DOI:** 10.1007/s40037-017-0381-x

**Published:** 2017-10-25

**Authors:** Eric Mercier, Pier-Alexandre Tardif, Marcel Émond, Natalie Le Sage

**Affiliations:** 1 Axe Santé des Populations et Pratiques Optimales en Santé, Unité de recherche en Traumatologie—Urgence—Soins Intensifs, Centre de recherche du CHU de Québec, Université Laval, Québec, Canada; 2 Centre de recherche sur les soins et les services de première ligne de l’Université Laval, Québec, Canada; 3 School of Public Health and Preventive Medicine, Monash University, Melbourne, Victoria Australia; 4 Centre d’excellence sur le vieillissement, Centrede recherche sur les soins de première ligne de l’Université Laval, Québec, Canada

Shortly after publishing our first article, we (EM, PAT) started receiving daily electronic invitations to submit additional manuscripts to unfamiliar journals and present at questionable conferences. Unfortunately, our experience is not unique. Considering the intense pressure to publish during medical training, limited knowledge of predatory publishing entities, and lack of local institutional policies to guide trainees’ responses to these flattering invitations, this trend is especially concerning.

Publishing in medical journals and presenting at health conferences are valued accomplishments across the continuum of medical education. For example, the number of publications is often used as a metric of productivity and postgraduate trainees are expected to possess lengthy bibliographies to compete for positions, promotions, and grants. Specific to novice researchers, their number of research accomplishments is associated with successful applications in competitive residency programs [1]. However, despite the numerous barriers to publishing in well-established journals (e. g., costs, rejection rates, delays, etc.), trainees are facing an unprecedented pressure to publish.

From our experience, there is a lack of awareness regarding publication ethics and the nature of predatory entities. Personally, we have never been taught about this phenomenon. Furthermore, our discussions with other trainees and their teachers has confirmed our assumption that this is a gap in medical education.

Formal academic institution policies and code of conduct promoting publication in reputable journals over potential predatory publishers are rare [2]. These documents should reflect the institutions’ ideal of academic excellence and be endorsed by the research community. Such policies could raise researchers’ awareness and discourage unethical behaviours using a structured process to evaluate the use of predatory entities and its potential consequences.

Finally, we are aware of junior colleagues who have published in questionable journals or submitted manuscripts that were never published after having transferred their copyrights. As predatory entities who prioritize profit over scientific value continue to expand, medical trainees will increasingly be targeted.

Concerted actions are required to counteract this phenomenon and protect trainees. For instance, publication ethics should be taught concomitantly with the medical student’s introduction to scientific literature and methodology. Additionally, structured educational programs should incorporate training to enhance trainees’ ability to appraise journal and publisher credibility (e. g., using guidelines promoted by the Committee of Publication Ethics) [3]. Clinical teachers and researchers should also work together to mentor students in interpreting, producing and presenting research results ethically. Finally, we think development of institutional policies is essential and we intend to participate in the development of one at our institution.
